# Does Provider Bias Affect Choice of a Facility for Family Planning Services by Women in Urban Senegal?

**DOI:** 10.1111/sifp.12181

**Published:** 2022-01-26

**Authors:** Ilene S. Speizer, David K. Guilkey, Jennifer Winston, Lisa M. Calhoun

**Affiliations:** ^1^ is Research Professor, Department of Maternal and Child Health and Faculty Fellow, Carolina Population Center University of North Carolina at Chapel Hill 123 W. Franklin St. Chapel Hill NC 27516 USA; ^2^ is Boshamer Distinguished Professor, Department of Economics and Faculty Fellow, Carolina Population Center University of North Carolina at Chapel Hill 123 W. Franklin St. Chapel Hill NC 27516 USA; ^3^ are Research Associates, Carolina Population Center University of North Carolina at Chapel Hill 123 W. Franklin St. Chapel Hill NC 27516 USA

**Keywords:** provider bias, quality of care, Senegal

## Abstract

Few studies to date have determined the effect of provider bias based on age, parity, and marital status on women's method and facility choice. Using data from women using modern methods in six cities of Senegal and a facility survey that included a facility audit and provider interviews, we undertake conditional logit analyses to determine whether women's choice of a family planning facility is associated with provider bias at the facility, controlling for other facility characteristics (e.g., size, sector, and number of methods available). We find that women bypass facilities where there is greater provider bias to attain their current family planning method. Women also bypass facilities of lower quality. This is the first study to demonstrate the effects of provider bias on women's contraceptive seeking behaviors and suggests the importance of training providers to reduce age and parity bias that affect access to a full range of methods and facilities for all women.

## INTRODUCTION

Provider bias in family planning services, originally defined by Shelton, Angle, and Jacobstein (1992) as one of six medical barriers to family planning, has been documented to take place in numerous countries, types of facilities (hospitals, health centers, health posts, and pharmacies), and sectors (public and private) (Calhoun et al. 2013; Tumlinson, Okigbo, and Speizer 2015; Schwandt, Speizer, and Corroon 2017; Sidze et al. 2014; Speizer et al. 2000; Sieverding et al. 2018). Provider bias often is manifested through providers refusing any (or all) services to a client based on her (or his) demographic characteristics; offering some methods to clients based on pre‐conceived perspectives of what is safe and appropriate for different types of clients; and introducing additional hurdles to contraceptive provision that are not based on medical or clinical guidance (Solo and Festin 2019). For example, some providers may refuse family planning services or specific methods to unmarried women or others may require partner consent; these restrictions are likely a reflection of social norms in the providers’ communities that reject nonmarital sex or consider the husband as the main decision‐maker (Sidze et al. 2014; Starling et al. 2017). Further, providers may choose not to offer some methods to young, unmarried, or nulliparous women based on misconceptions about side effects or subsequent fecundability and concerns about promiscuity among young or unmarried users (Barden O'Fallon et al. 2021; Solo and Festin 2019; Starling et al. 2017).

Much of the literature on provider bias has focused on documenting that it exists using provider surveys, mystery clients, or qualitative data collection with clients or providers (Calhoun et al. 2013; Mchome et al. 2015; Schwandt, Speizer, and Corroon 2017; Sidze et al. 2014; Speizer et al. 2000; Schuler and Hossain 1998; Tumlinson, Okigbo, and Speizer 2015). Studies using provider surveys demonstrate that some providers report minimum age requirements for hormonal and long‐acting methods; parity requirements (usually at least one child) for provision of injectables, implants, and intrauterine devices; and marital status or consent requirements for provision of some methods (Calhoun et al. 2013; Schwandt, Speizer, and Corroon 2017; Sidze et al. 2014; Speizer et al. 2000; Tumlinson, Okigbo, and Speizer 2015). Service provision approaches may be influenced by provider training as demonstrated in a study from Nigeria that found that health facility providers (nurse/midwives or community health extension workers) who had in‐service training were less likely to impose marital status biases than their counterparts who did not receive in‐service training (Schwandt, Speizer, and Corroon 2017); no difference by training was found for pharmacists or drug shop providers.

In a study that included provider data from Senegal, the site of this study, Sidze et al. (2014) demonstrated that more than two‐fifths of providers reported minimum age biases for pills and injectables in public hospitals, health centers and health posts, the most common sources of these methods. For implants, more than two‐fifths of providers restricted the method based on minimum age requirements in hospitals and health centers; only one‐third of providers reported the same in health posts, where about one‐third of implant users received their method. Further, the authors demonstrated that nurses were more likely to impose age restrictions (in public sector facilities where most clients obtain their methods) than doctors or midwives and male providers were more likely to impose age restrictions on injectable access than female providers (Sidze et al. 2014).

Qualitative studies have been used to show provider bias and providers’ opinions and beliefs that guide their provision of family planning services (Mchome et al. 2015; Sieverding et al. 2018). In a study that triangulated mystery client visits with in‐depth interviews in South West Nigeria, Sieverding et al. (2018) demonstrated that providers often tried to discourage young/unmarried clients from using hormonal methods, and in‐depth interviews clarified that this was related to the providers’ belief that these clients are better off abstaining from sex. Further, providers also thought that young clients were likely to be having irregular sex and therefore condoms and emergency contraception (EC) were considered more appropriate methods for them (Sieverding et al. 2018). In another mystery client study from Tanzania, Mchome et al. (2015) demonstrated that providers treated adolescent clients as if they should not be having sex or using contraception until they are married.

Although earlier studies demonstrated the existence of provider bias, the implications of this bias on women's access to and use of health facilities and contraception is not well‐documented (Solo and Festin 2019). The challenge with assessing the role of provider bias on actual contraceptive use is a lack of relevant data to link provider‐based bias to women's method or facility choice. This study begins to explore this issue by examining users of modern contraception in urban sites of Senegal and determining the association between facility characteristics related to quality and provider biases and women's choice of where to obtain their contraceptive method. We hypothesize that, controlling for other attributes of facilities related to quality of services, women will choose facilities with less provider bias. An assumption of this analysis is that women know about facility attributes and potential biases and are choosing facilities based on this prior knowledge. This is not an unreasonable assumption given that earlier studies have shown that perceptions of health care quality are often informed by social networks and information exchange (Hanefeld, Powell‐Jackson, and Balabanova 2017).

## METHODS

### Study Context: Senegal

This study uses secondary data from Senegal, an early commitment maker to the FP2020 global family planning initiative. Just prior to the launch of FP2020, Senegal had a prevalence of modern method use among women in union in 2010–2011 at 12.1 percent (ANSD and ICF 2012) and made a commitment to increase it to 45 percent by 2020.^1^ At the time of this study (2015) the total fertility rate in Senegal was 4.9 births per woman; the corresponding value was 3.5 in urban areas and 6.1 in rural areas (ANSD and ICF 2015). In 2015, among women married or in union, modern method use was 21.2 percent, with higher levels in urban (30 percent) than rural areas (15 percent). Among women aged 15–49 years in Senegal in 2015, 65 percent were married or living with a partner and the median age at marriage among women aged 20–49 was 19.7 years (ANSD and ICF 2016). Modern contraceptive use among women in union was highest among women aged 30–44 years and lowest among women in union aged 15–19 years. The main contraceptive method used in Senegal was injectables, followed by similar levels of implant and oral contraceptive pill use. Senegal's health system includes public sector and private sector facilities. The public health system has hospitals at the central and regional levels and health centers and health posts that are accessible to all. It is notable that in Senegal, 80 percent of private sector facilities are located in Dakar. The private sector consists of hospitals, clinics, pharmacies, and retail outlets. Nationally, 86 percent of contraceptive users source their method from the public sector, though there are differences by geography, with 81 percent of urban and 94 percent of rural users sourcing their method from the public sector.^2^

### Data Sources and Sample

Data for this study come from six urban sites in Senegal and were collected as part of the Measurement, Learning and Evaluation (MLE) project. The MLE project was tasked with evaluating the Bill & Melinda Gates Foundation funded Senegal Urban Reproductive Health Initiative and to do so, collected longitudinal data from women and health facilities as well as a survey of providers at each of three survey rounds (baseline, midterm and end line). This analysis utilizes end line data, collected in 2015, from women, health facilities, and providers. Individual women were matched to the facilities they visited to receive family planning services. End line data were used because there were more users at end line compared to the earlier rounds, we were able to match more women to the facility that they visited for their method, a much larger number of facilities participated in the facility survey, and it is the most recent.

Details of the longitudinal sample collected as part of the MLE project can be found elsewhere (Benson et al. 2018) but briefly, at baseline, the study included a representative sample of women aged 15–49 from each of six cities (Sidze et al. 2014). Four years later, these same women were surveyed at their household or if they moved, at their new location (end line). Geographic positioning system (GPS) coordinates were collected at the centroid of each cluster or at the woman's home if she moved. At each survey round, women were asked about their demographics, family planning and sexual and reproductive health attitudes and behaviors, and exposure to family planning programming, among other areas. At end line, 6,927 women were surveyed; this represents 84 percent of the baseline sample (Benson et al. 2018). This analysis focuses on the women who were using a modern method at end line and could be matched to the health facility where they received their method. A total of 1,727 women (24.9 percent of the end line sample^3^) were using a modern method at end line. Modern methods include pills, condoms, injectables, implant, intrauterine device (IUD), female condoms, Standard Days Method (SDM), and lactational amenorrhea method (LAM). These last three methods are considered “other modern methods” for this analysis (see Table 1). Of the women using a modern method at end line, we were able to match 1,266 of them (73.3 percent) to the health facility where they reported they received the method. The women who did not match either (a) received their method from a pharmacy or drug shop (e.g., condoms) or were using a nonfacility‐based modern method (e.g., SDM or LAM) (n = 114); (b) visited a facility that was outside the study cities (n = 12); or (c) reported a facility that we were not able to match to our facility sample (n = 335). The 1,266 women who we were able to match to a facility represent the main analysis sample for this study, however, the full sample of respondents was used to test for selection bias using methods described below.

**TABLE 1:** Characteristics of women in Senegal end line survey

			Using a modern method			
	Full sample		All users		Users matched to any health facility	
	Percentage	N	Percentage	N	Percentage	N
Age						
<26	30.7	2,128	16.2	249	16.2	179
26+	69.3	4,799	83.8	1,285	83.8	929
Union status						
Not in union	39.6	2,742	5.7	87	5.8	65
In union	60.4	4,185	94.3	1,446	94.2	1, 044
Education						
None/Quaranic	18.4	1,271	19.5	298	20.8	230
Primary	44.4	3,076	54.3	833	54.3	601
Secondary	27.5	1,905	22.3	342	22.1	245
Higher than secondary	9.7	673	3.9	60	2.8	31
Muslim	90.4	6,263	93.8	1,438	94.6	1,048
Wealth						
Lowest	21.7	1,506	22.5	345	20.6	228
Low	19.8	1,373	21.8	335	23.9	265
Medium	19.2	1,329	20.4	313	21.4	237
High	20.3	1,403	19.3	296	20.9	232
Highest	19.0	1,315	16.0	245	13.3	147
City of residence						
Dakar	41.4	2,864	37.2	571	33.9	376
Pikine	10.7	737	10.7	165	11.3	125
Guédiawaye	11.0	762	12.3	188	15.0	166
Mbao	22.7	1,572	23.9	366	24.4	270
Mbour	6.8	468	9.4	144	8.7	96
Kaolack	7.6	523	6.5	100	6.9	76
Using a modern method	22.1	1,534	NA	NA	NA	NA
Method used						
Condom	NA	NA	5.6	86	0.2	2
Pill			21.2	326	20.8	231
Injectable			34.5	528	38.2	424
LARC (implant/IUD)			38.2	585	40.7	451
Other modern			0.6	8	0	0*

NOTE: Weighted number of observations and percentages shown. Unweighted number of observations is 6,927 in the full sample, 1,727 for all users, and 1,266 for users linked to a facility. Other modern methods: female condoms, Standard Days Method, and lactational amenorrhea method.

* Significant difference between full sample of users and those with facility information at p < 0.05.

Table 1 presents the characteristics of the full sample of respondents at end line, the full sample of contraceptive users at end line and the analysis sample. As seen in Table 1, users are older than the full sample; however, there are no significant demographic differences between users matched to a facility and users we were unable to match. Not surprisingly, users are significantly more likely to be currently in union than the full sample (94 percent vs. 60 percent). No difference is observed in union status by whether we were able to match the users to a facility. Compared to the full sample, those who are using were slightly less educated than nonusers; this might be reflective of the user sample being older. No significant difference is observed between the user sample and the full sample in terms of religion, wealth, and city of residence. In the full sample of women, 22 percent were using a modern method at end line. Among all users, 38 percent of the sample were using long‐acting reversible contraceptives (LARCs; implants and IUD), followed by 34.5 percent using injectable, and 21 percent using pills. About 6 percent of women were using condoms (5.6 percent) or another modern method (female condom, SDM, LAM). In the analysis sample, the condom users and the other modern method users were dropped as they did not visit a health facility the last time they received their method; the exception is two condom users who reported a facility where they obtained their method (see Table 1). As shown in Table 1, the distribution of the methods used between the analysis sample and the full sample of users is significantly different reflecting that condom users are generally not included in the analysis sample given that they received their condoms from a nonfacility source.

At the time of the end line survey, the MLE team also collected data at all public and private health facilities offering reproductive health services in the six study cities. In total, facility data were collected from 249 of 262 identified facilities in the six cities. Of the facilities not interviewed (n = 13), three were destroyed/moved/or not functioning, one was closed, three refused to participate, and the remaining (n = 6) did not offer family planning or sexual and reproductive health services. As part of the facility survey, we undertook a facility audit, provider surveys, and exit interviews. For this analysis that examines facility choice based on characteristics of the facilities, we use information from the facility audit to inform some of the facility quality variables and information from the provider surveys to create the provider bias variables. At each facility, a facility administrator was asked questions about the facility including the age of the facility, the number of staff, outreach activities, services offered, and family planning method provision, among other things. In addition, we obtained a GPS point at each facility; this was used to create the distance from the center of the women's household clusters to each facility, or for women who moved, from her new household location to each facility.

In each facility, up to four providers were surveyed about their services and practices in the facility. In larger facilities, four providers who offered family planning and/or sexual and reproductive health services were randomly selected to participate and in smaller facilities (with four or fewer providers), all providers were approached for interview. In total, 781 providers were surveyed across the sites and within a site, one to four providers were surveyed. Notably, one of the surveyed facilities did not provide provider data for the provider bias questions described below and therefore the final facility‐level sample is 248 facilities for the analysis.

All study procedures, consent materials, and data collection tools for the household and facility‐based surveys were reviewed and approved by the Institutional Review Board at the University of North Carolina at Chapel Hill and the Comité National d'Ethique pour la Recherche en Santé in Senegal. All women and providers provided written consent to participate in the survey.

### Variables

As described above, we are modeling the choice of a facility among women who are using a modern method of contraception. The key outcome variable is facility choice, that is the specific facility where each woman received her method. The key independent variables in this analysis relate to the characteristics of the facilities (see Table 2). Among the independent variables measured through the facility audit, we include the type of facility (public vs. private), facility age (in years), whether the facility is open seven days a week, the number of midwives at the facility, the number of modern methods available at the facility, if the facility does community talks or outreach, if the facility has an assistant or social worker, the number of family planning clients (new and continuing) in the last 30 days, and if the facility had a stock out of pills or injectables in the last year.

**TABLE 2:** Characteristics of facilities where women get family planning methods at end line

	All health facilities^a^		Nearest health facility for FP^b^		Chosen health facility for FP^2^	
Type of facility (%)	Value	Number	Value	Number	Value	Number
Public facility (clinic/hospital)	75.0	186	81.4	903	96.1*	1,066
Private facility (clinic/hospital)	25.0	62	18.6	206	3.9*	43
Average facility age (years and range)	26.4	0–115	25.4	0–95	29.9*	2–115
Average distance (average km and range)	NA	NA	0.40	0.02–5.14	1.94*	0.02–21.17
Open seven days a week (%)	59.7	148	57.2	634	63.5	704
Average number of midwives (n and range)	2.6	0–24	2.7	0–22	4.6*	0–24
Average number of clients last 30 days (n and range)	119.0	0–618	172.0	0–618	215.0*	0–618
Facility does community talks/outreach (%)	43.5	100	50.0	554	61.2*	678
Facility had a social worker (%)	13.3	33	9.1	101	28.4*	314
Average number of methods available (n and range)	7.2	0–9	7.6	0–9	8.4*	0–9
Stock out of pill or injectable in the last year (%)	4.8	12	3.5	38	3.0	33

^a^ Number of health facilities is 248.

^b^ Results based on weighted sample of women matched to a facility; weighted n's presented. Unweighted number of women is 1,266.

NA, distance is measured from a respondent's cluster to each facility so in the facility sample, there is no average distance.

* Significant difference between nearest and chosen facilities at p < 0.05.

We also include distance to each facility from the women's household clusters for women who did not move between baseline and end line or from the women's homes for women who moved between the survey rounds. Of the 248 facilities included, 201 are in the four cities in the greater Dakar area (Dakar, Guédiawaye, Pikine, and Mbao), 27 are in Kaolack and 20 are in Mbour. In the greater Dakar area, the farthest any respondent lived from a facility was less than 25 km and so we assumed that all 201 facilities were possible choices for each respondent. Distances were much shorter for Kaolack and Mbour and we assumed that the choice set for each woman was all facilities in her city. Allowing respondents such large choice sets seems reasonable since a respondent may go to a facility that is close to work or a market as opposed to only facilities close to home. Given the density of facilities in these urban settings, the median distance a woman traveled was only 0.7 km but the maximum distance traveled was over 21 km and 65 percent of the women bypassed the closest facility to her home in order to visit a facility of higher quality. In addition, in earlier work (Cronin, Guilkey, and Speizer 2019), we found that restricting the choice set that a respondent faces to only facilities within 5 km of the respondent's community can lead to misleading results.

Table 2 provides the characteristics of the facilities in the facility analysis sample (n = 248), the nearest facilities to the respondents’ place of residence, and the chosen facility among women using a modern method who matched to a facility. The majority of the facilities in the sample are public sector clinics or hospitals (75 percent) and the remainder are private sector facilities (25 percent). Notably, among the facilities selected by the women, 96 percent of them are public sector facilities, whereas among the nearest facilities 81 percent were public; this difference is significant at p < 0.05. On average, facilities were open 26.4 years (range 0–115). Among the facilities selected by the women, they were open a slightly longer period (29.9 years); this was longer than the time open of the nearest facilities. About 60 percent of facilities were open seven days a week and a similar percentage of women (63.5 percent) chose facilities that were open every day. Table 2 shows the average number of midwives in the facilities (2.6 among all facilities and 2.7 in the nearest facilities) and that women chose facilities that were larger and had more midwives on average (4.6). This is also represented in the average number of clients in the last 30 days whereby women chose facilities with a greater average number of clients than in the sample of all health facilities and the nearest health facilities. Women were more likely to choose facilities that offered community talks and had an assistant or social worker as compared to the full sample and the nearest facilities. On average, facilities had 7.2 modern methods (out of nine) and women chose facilities that had more modern methods available (8.4); this was significantly higher than the number of methods available at the nearest facilities (7.6). Finally, while stock outs of pills and injectables were rare across the facilities (4.8 percent), women chose facilities with fewer stock outs of these methods.

Also included in the set of facility characteristics were several different measures of provider bias measured based on the provider surveys. Table 3 presents the survey questions about provider bias as well as the methods asked about, the response options, the coding strategy, and the approach to aggregating the information. All the bias measures were asked separately about each method (male condom, pills, injectable, IUD, and implant). Parity bias is coded as “yes” if a provider said that there is a specific number of children that a client had to have to get any of the methods. Minimum age bias is coded “yes” if a provider said there was an age over 15 years that the client had to be to receive any of the methods (e.g., bias if provider said client had to be at least 17 years to receive an implant). Maximum age bias is coded “yes” if a provider said the client had to be under some age (less than 49) to receive any of the methods (e.g., bias if provider said client had to be under 35 years to receive an IUD). Marital status bias is coded “yes” if a provider reported that a client had to be married to receive any of the methods. Likewise, consent bias is coded yes if the provider required consent from someone else to receive any of the methods. Not shown in Table 3 is any bias that includes any age bias, parity bias, marital status bias, or consent bias and any age bias that is based on reporting a minimum or a maximum age bias. Responses from individual providers (up to four per facility) were aggregated at the facility level for each bias variable across methods to get the average value for each type of bias for the facility (see Table 3).

**TABLE 3:** Questions and coding strategy for bias measures

	Methods asked about	Response options	Coding strategy	Aggregation
For each method offered by provider, ask the following question:				
What is the minimum age below which you will not offer or counsel the method?	All questions about the following methods: Oral contraceptive pill Injectable Male condom IUD Implant	Age _______ No minimum Do not know	Coded 1 (bias) if age given is 16 or older; otherwise, coded as zero (no bias)	Created variable for each bias measure as whether each provider reported a bias for any method by type of bias At the facility‐level: assessed the percent of providers surveyed (up to 4) that reported any bias
What is the maximum age over which you will not offer or counsel the method?		Age _______ No maximum Do not know	Coded 1 (bias) if age given is an age between 16 and 48; otherwise, coded as zero (no bias)	
Is there a minimum number of children that a woman must have before you will offer or counsel her on the method? What is that minimum number of children?		Yes—if yes, number ____ No Do not know	Coded 1 (bias) if having any children is a requirement for receiving a method; coded zero otherwise (no bias)	
Do you require partner consent before offering or counseling the method?		Yes No	Coded 1 (bias) if partner consent is required; coded zero otherwise (no bias)	
Would you offer or counsel the method to a person who is not married?		Yes No	Coded 1 (bias) if client is required to be married to receive the method; coded zero otherwise (no bias)	

NOTE: Questions about bias toward other methods less used (progestin‐only pills, female condom, emergency contraception, Standard Days Method, and female and male sterilization) were included but are not part of the bias measures.

### Analysis Approach

The method of estimation that we use is conditional logit analysis (McFadden 1974; Train 2009), which is a standard model that is used for discrete choice analysis in which a respondent is choosing among an unordered categorical set of outcomes (facilities in this case). A similar approach was used by Elewonibi et al. (2020) in their analysis of distance‐quality trade‐off for choice of a family planning facility in Tanzania. We use information about the attributes of the facility that each respondent actually chose along with the attributes of all the facilities that she did not choose. Individual level variables such as age and education are not included because they do not vary across the choice set. A major advantage of the conditional logit model is that, unlike multinomial logit, the number of parameters to estimate does not increase with the number of facilities in the choice set. It only increases as you add facility attributes to the model specification. The basic form of the model is:

(1) $$U_{ij}=X_{ij1}\beta_{1}+X_{ij2}\beta_{2}+\cdots +X_{ijK}\beta_{K}+\varepsilon_{ij},$$

where *U_ij_* is the utility that individual *i* = 1,2,…N receives from facility *j* = 1,2,…,*J*. The *X_ijk_* (*k* = 1,2,…,*K*) represent facility attributes that include items such as indicators for whether or not the facility is open all hours, the number of midwives, and the provider bias variables. Also included in *X* is the distance the facility is to each community where respondents live. The β’s are unknown parameters to be estimated and can be thought of as the weight that respondents put on each of the observed attributes of the facilities in determining their utility. We view distance as our “price” variable, and we expect distance to have a negative effect on utility and so the β should be negative. In other words, women will prefer facilities that are closer rather than further away and will only choose a more distant facility if it has a quality attribute (less bias for example) that the respondent values.

If we make the standard assumption that an individual wants to maximize utility, then the probability that individual *i* chooses facility *j* is:

(2) $$P_{ij}=\mathrm{Pr}\left(U_{ij}\geq U_{ik}\right)\;\text{for}\;\text{all}\;k\neq j.$$

Specifying these probabilities as functions of the observed attributes and the unknown β’s allows us to use maximum likelihood methods to estimate the β’s. However, as should be clear from equation (2), if we multiply the utilities of all choices by the same positive constant, the probabilities for each choice are not altered because the ordering of the utilities would not change. This means that the estimated β’s are all scaled by this same unknown positive constant. As a result, we can only interpret coefficients in terms of whether the corresponding variable has a positive or negative effect on a respondent's utility and whether or not the effect is significant because the positive constant would divide out when we form the *z* ratio (the estimated coefficient divided by its standard error). What is often done to remove the scale factor and increase the comparability is to present marginal effects. However, marginal effects are not informative here because of the large number of facilities that the individuals choose between. The largest number of individuals that report that they received their method from one facility is only 56 and the median number of individuals that report a particular facility is six. As a result, all marginal effects of the independent variables on the use of a facility are small. However, it is straightforward to interpret the estimated coefficients in terms of tradeoffs with the distance variable (our proxy for price). Therefore, in addition to presenting the model coefficients, we also present a “willingness to pay” or alternatively “willingness to travel” to obtain the method at a facility with less provider bias.

Willingness to pay (or willingness to travel) is simply the ratio of two coefficients where the coefficient of distance is in the denominator:

(3) $$WTP_{k}=\frac{{\hat{\beta }}_{k}}{{\hat{\beta }}_{1}},$$

where ${\hat{\beta }}_{1}$ is the estimated coefficient for distance and ${\hat{\beta }}_{k}$ is the estimated coefficient for some other facility attribute. In this case, we present the ratio of the bias coefficient to the distance coefficient to provide the average distance in kilometers that a woman is willing to travel to avoid a facility with each type of bias. This means that the willingness to pay measures can be directly compared for the separate regressions with different bias measures since forming the ratio removes any scale factor.

Reporting the results in terms of willingness to pay (or travel) is the standard way to help with the interpretation of the results from discrete choice models. See, for example, Train (2009) and Cronin, Guilkey, and Speizer (2019). The second paper is of particular relevance since it examines the willingness to pay for attributes of facilities for maternal and child health services in terms of distance traveled. There is also a Stata routine (WTP) that is available and we used it to calculate confidence intervals for the estimated willingness to pay measures (see Hole 2007).

Our original plan was to include all the bias measures in the model at the same time and estimate a single model. As one might expect, this introduced a lot of collinearity into the results. We therefore ran models where each bias measure was included on its own along with the complete set of facility attributes. Since there were seven bias measures, there were seven models run with the only difference being which bias measure was included (minimum age bias; maximum age bias; parity bias; marital status bias; consent bias, any age bias, and any bias). Also, to determine if there were distinctions in facility choice between younger and older women, we ran models by age group (<26 and 26+) and compared model results by age group and type of bias in a summary table. Note that because only a small number of users in urban Senegal were young, we selected the younger age group to include age 25 years and under, in case there was age heaping on age 25. In total, there were only 175 young users (unweighted number of observations) in this sample (contact the first author for the age disaggregated multivariate results). All descriptive results that focus on women used women‐level weights that adjusted for nonresponse and sampling at the city levels.

A final issue that must be addressed is that the set of 1,266 respondents who were modern users who could be matched to a facility were self‐selected and this could introduce bias. There are two approaches for correcting for selection bias: selection on unobservables or selection on observables (see Moffitt, Fitzgerald, and Gottschalk 1999 or Wooldridge 2010). Since selection on observables tends to yield more stable results, we adopted that strategy. Its steps were to first use the full sample of women minus the 12 women who went to a facility outside their city and define a four category variable: modern users matched to a facility (1,266), modern users who went to a pharmacy (114), modern users that could not be matched to a facility (335) and nonusers (5,200). We then estimated a multinomial logit model using the four categories as the dependent variable and a large set of individual characteristics such as age, education, city of residence, and number of living children as independent variables. The multinomial logit results were then used to predict the probability that a woman was a modern user matched to a facility and the inverse of this probability is the inverse probability weight that was used for a weighted conditional logit model that corrects for selection bias. The estimated coefficients were then used to test whether results corrected for selection were significantly different from the results not corrected for selection. Since we failed to reject the null that the selection corrected results were significantly different from the uncorrected results, we reported uncorrected results below. More details on these methods can be found in Cronin, Guilkey, and Speizer (2019); contact the first author for these specific results.

## RESULTS

The provider bias summary statistics are presented in Table 4 and represent the average percentage of providers who reported each type of bias for all methods by type of facility. Overall, age bias is more common than the other types of bias. On average, 23.8 percent of providers reported a minimum age bias for one or more methods. Further, 21.5 percent of providers reported a maximum age bias and when aggregated, 31.4 percent of providers across facilities reported any age bias. Age bias appears more common in private than public facilities; however, women were more likely to get their methods from public sector facilities, as shown in Table 2. The other types of bias are less common. Parity bias was found among 13 percent of providers and consent bias and marital status bias were found among around 9 percent of providers. Aggregating across the types of bias, we find that overall, 39.5 percent of facilities had any provider that imposes a bias on use of a method; the values are similar for public and private facilities.

**TABLE 4:** Average proportion of providers reporting bias by type of bias and type of facility (n = 248 facilities)

	Minimum age bias	Maximum age bias	Parity Bias	Marital status bias	Consent bias	Any age bias	Any bias
Type of facility							
Public facility	22.7%	20.3%	13.5%	7.7%	7.4%	31.1%	39.5%
Private facility	27.2%	25.0%	12.6%	13.6%	16.1%	32.3%	39.7%
Any facility type	23.8%	21.5%	13.3%	9.2%	9.5%	31.4%	39.5%

The multivariate conditional logit coefficients and standard errors for the full models are presented in Table 5 by type of age bias and parity bias. Note that models by age group (<26 and 26+) are available from the first author; given that most of the users were in the older age group, the all age group models are similar to results for the older age group. All models include a large number of observations since an observation represents a woman/facility pair and women are paired with each facility in their city. In each model, the minimum age, maximum age, and parity bias measures are negative and significant. For example, in Table 5 in the model for minimum age bias, we see that women were significantly less likely to choose a facility that has minimum age bias than a facility without such bias for their current contraceptive method (*β* = 0.‐5014; SE = 0.1008; p ≤ 0.001). Further, we see consistently across the models that distance is negative suggesting that women were less likely to choose facilities that were further away. Some other consistent results (consistent across the other bias models as well) are that women were less likely to choose private sector facilities as compared to public sector facilities. Further, women tended to choose facilities that were larger (i.e., had a large average number of clients in the last 30 days). Finally, women were significantly more likely to choose facilities that were older, had an assistant or social worker, and had more methods available than facilities that are younger, did not have an assistant or social worker and had fewer methods available.

**TABLE 5:** Multivariate coefficients (SE) for choice of a facility for family planning with age bias and parity bias measures

	Minimum age bias		Maximum age bias		Parity bias	
	Coef.	SE	Coef.	Coef.	Coef.	SE
Bias measure	−0.5014	0.1008***	−0.6214	0.1223***	−0.4755	0.1503**
Average distance	−1.0003	0.0646***	−1.0012	0.0645***	−0.9995	0.0646***
Open seven days a week	−0.0227	0.0706	−0.0154	0.0706	−0.0328	0.0703
Average number of midwives	0.0082	0.0107	0.0017	0.0105	0.0090	0.0108
Average number of clients last 30 days	0.0017	0.0003***	0.0019	0.0003***	0.0018	0.0003***
Facility age	0.0058	0.0015***	0.0060	0.0015***	0.0058	0.0016***
Facility does community talks/outreach	0.1391	0.0681*	0.1212	0.0686+	0.1031	0.0705
Facility had a social worker	0.4414	0.1215***	0.4456	0.1194***	0.4491	0.1190***
Average number of methods available	0.0796	0.0272**	0.0828	0.0267**	0.0761	0.0268**
Stock out pill or injectable in the last year	−0.1156	0.1791	−0.1720	0.1832	−0.2376	0.1889
Private facility	−1.6028	0.2104***	−1.5627	0.2070***	−1.6515	0.2088***
Number of observations in model	152,681		152,681		152,681	

NOTE: Sample size for analysis is the total number of facilities that the sample of women could potentially access in their city. The coefficients show whether or not the corresponding variable is given positive or negative weight when choosing a facility. The standard errors are cluster corrected for multiple observations on the same respondent since each respondent is linked to all facilities. ^+^p ≤ 0.10,

* p ≤ 0.05, ^**^p ≤ 0.01, ^***^p ≤ 0.001.

Table A1 presents similar models for the marriage bias, consent bias, any age bias, and any bias measures. With the exception of consent bias, all of the bias measures are negative and significant, as above. In the model for consent bias, we see a positive and significant coefficient (p < 0.05) indicating that women were more likely to choose a facility that requires consent than a facility that does not require consent. Finally, for the models with the any age bias and any bias, we find that women were less likely to visit a facility with more bias. For the other facility characteristics, including distance, we find similar effects of the variables on facility choice as discussed in the age bias models previously.

In Table 6, we present summary results across the minimum age, maximum age, and parity bias measures and include the summary results by age group as well. As mentioned above, it is well‐known that logit coefficients cannot be compared across models. However, ratios of coefficients can be directly compared. Therefore, as explained earlier, we divided each of our bias measures by the distance coefficient that was highly significant and negative in all estimations. The ratio is interpreted as the respondents’ “willingness to pay” or “willingness to travel” to obtain a particular facility attribute. In this case, since most of the significant bias coefficients are negative, the ratios indicate how far in kilometers a respondent is willing to travel to *avoid* a particular bias. For example, in the all age sample, women will travel 0.50 (table value is 0.5012) additional kilometers to avoid a facility where there is minimum age bias and about 0.62 additional kilometers to avoid a facility with maximum age bias. Notably, in the Dakar region, there are numerous facilities available to women, so while these distances are not far, they do represent bypassing of facilities where some of these biases may exist. For all types of bias, except consent bias (see Table A2), women are bypassing facilities and willing to travel from a quarter to two‐thirds of a kilometer further to avoid a facility with bias. Summary results by age group presented in Table 6 are similar to the all age results; however, for the younger age group (<26 years) the parity bias results do not attain significance at p ≤ 0.10.

**TABLE 6:** Summary comparison of results (willingness to travel) from models of minimum age bias, maximum age bias and parity bias presented in Table 5

	Willingness to travel in kilometers for avoiding bias (95% confidence interval in parentheses)		
Group	Minimum age bias/distance	Maximum age bias/distance	Parity bias/distance
All ages	0.5012 (0.2954, 0.7070)	0.6203 (0.3867, 0.8546)	0.4757 (0.1771, 0.7744)
<26	0.4194 (−0.0257, 0.8644)	0.5867 (0.0731, 1.1004)	0.3155 (NS) (−0.2910, 0.9220)
26+	0.5094 (0.2816, 0.7373)	0.6156 (0.3554, 0.8759)	0.5048 (0.1699, 0.8397)

NOTE: Models control for: distance, whether open all days of the week, number of midwives, number of new or continuing family planning clients, facility age, whether the facility does community talks, whether there is a social worker at the facility, the number of methods available, if there has been a stock out of pills or injectables in the last year, if it is a private facility, and the bias measure. (NS) indicates that the result is not significant at p ≤ 0.10.

## DISCUSSION

This study begins to fill a gap in our understanding of how provider bias is associated with access to and use of contraception. Prior studies have simply documented that provider bias exists, but this study examines whether women's choice of a facility for their family planning method was associated with greater (or less) provider bias at a facility. We hypothesized that, controlling for facility attributes related to quality of services, women choose facilities with less provider bias to obtain their family planning method. The underlying assumption in this analysis is that women are aware of various facility attributes, including if providers are biased, and are weighing their choice of facility based on this prior knowledge. This is a realistic assumption given that previous studies have demonstrated that perceptions of health care quality are often informed by social networks and information exchange (Hanefeld, Powell‐Jackson, and Balabanova 2017). We examined provider age bias (minimum, maximum, and any age bias), parity bias, consent bias, and marriage bias toward provision of a modern method. By matching users of family planning in urban Senegal to the facility where they received their method and by having characteristics of all facilities accessible to women in their study city, we were able to undertake this unique analysis showing that bias was associated with choice of facility. Our results demonstrated that women were likely bypassing facilities where there was more age bias (minimum or maximum), parity bias, marital status bias, and any type of bias to get their method at a facility that had less of these types of bias. For example, women traveled up to 0.6 kilometers further to obtain their method at a facility with less age bias.

In our analysis, we found that consent bias was significant in the opposite direction (i.e., women choose facilities with consent bias). It is possible that this counterintuitive finding relates to the correlation between consent bias and private facilities; in models that removed the type of facility and size of facility, the consent bias result became negative and significant (i.e., women are less likely to choose facilities with consent bias). Further, the effect of marriage bias was not significant in the age‐stratified models (not shown). This null result may relate to the fact that most users in Senegal were in union and thus issues around marriage (and possibly consent) may be less salient to these women.

Our results demonstrate descriptively that biases were less common in public sector facilities and that the overwhelming majority of women were choosing public sector facilities. From our results, we cannot say whether this choice relates to less bias at public facilities or to the greater acceptability and lower cost of public sector services in urban Senegal.

These results contribute to the literature by demonstrating that providers reported various types of bias toward provision of contraceptive methods, as has been shown in other countries with quantitative data (Calhoun et al. 2013; Schwandt, Speizer, and Corroon 2017; Sidze et al. 2014; Speizer et al. 2000; Tumlinson, Okigbo, and Speizer 2015) and from mystery clients and qualitative data collection (Mchome et al. 2015; Schuler and Hossain 1998; Sieverding et al. 2018). An earlier study from urban Senegal using baseline data from the same project demonstrated that more than two‐fifths of providers reported minimum age biases for pills, injectables, and implants (Sidze et al. 2014). It is notable that in our sample of facilities surveyed four years later after the Urban Reproductive Health Initiative intervention had been implemented, provider bias was less commonly reported (about a quarter of providers reported any minimum age bias). Although we did not examine the impact of the program on provider bias, the lower levels of bias at the later period were suggestive of improvements in service quality and provision of services.

Recent reviews on provider bias have demonstrated that bias takes various forms (e.g., bias toward certain types of clients or methods), have discussed the causes or influences of bias, and have examined intervention strategies to address provider bias (Solo and Festin 2019; Starling et al. 2017). Our analysis is timely as it can help to fill the gap identified in these earlier reviews that do not examine how access to a full range of methods and sources of methods is affected by bias (Starling et al. 2017; Solo and Festin 2019). We show that provider biases were associated with choice of a facility and thus were associated with women's access to modern contraception.

Our study also demonstrates that women were frequently bypassing the nearest facilities and choosing facilities that are public sector, are larger, are older, do outreach, and offer more methods. This is like results from a recent study from Tanzania that examined bypassing behavior of women for contraceptive services and demonstrated that women were willing to travel further for family planning services at facilities that they perceived to be higher quality in terms of providing a range of methods, having fewer stock outs, and specializing in provision of contraception (Elewonibi et al. 2020). Similarly, studies of primary care and maternal health seeking behaviors have demonstrated that women bypassed the nearest facilities to visit facilities that they perceived to be of better quality in terms of services provided or provider competence (Bell et al. 2020; Kruk et al. 2009; Yao and Agadjanian 2018).

This study is not without limitations. First, since we focus on current users of modern contraception, we were not able to say whether provider bias affects adoption and use among women who were currently not using a method. If this is the case, these nonusing women may have sought a family planning method but were refused by a provider based on age, marital status, or parity; this is not captured in this analysis. Second, there were some users who we were not able to link to the facility where they received their method. If these women chose their source for reasons related to provider bias, this may affect the generalizability of our results. Third, while we set out to examine if there were differences in choices made related to provider bias between younger and older women, our urban Senegalese sample only had a small number of young users (under age 26), which limited our ability to examine them in more depth. Future analyses in another country where a larger number/proportion of young people use a modern contraceptive method and matched data are available could help to answer this question about whether younger (i.e., 15–19 or 15–24) and older women experience and respond to provider bias differently. Fourth, provider bias was self‐reported by the providers and it is possible that it is under‐reported should the providers feel that they should give some “expected” response; with the data available it is not possible to determine if under reporting is a concern in these data. Further, some providers may have been trained on provider bias as part of the intervention in the study cities. It was not possible to control for this specific training in this analysis. The data used in this study are from 2015, so changes in provider attitudes and behaviors may have happened since that time; that said, provider biases are based on social norms that are slow to change and thus we think the results are still relevant to scenarios where bias exists. Finally, we assume that women were aware of facility attributes and provider bias and were using this to inform their facility selection. Although we cannot validate this with the data available, we observed that women were bypassing facilities of lower quality, which suggests that they were making choices about where to seek their family planning services.

Notwithstanding these limitations, this analysis demonstrates important associations of provider bias with women's choice of where to obtain a family planning method. Women are bypassing facilities where provider bias may exist and choosing to visit facilities with less provider bias. This is particularly true for age and parity biases. Notably, in this Senegalese urban sample, women are also choosing facilities based on other characteristics including choosing public sector facilities, larger facilities, and facilities that do outreach. To better understand how provider bias affects women's complex decision‐making, future studies may need to undertake qualitative data collection that asks women (users and nonusers) to rank various factors that affect their contraceptive use and facility choice decision‐making, including provider engagement and treatment. Based on what is learned, it will be possible to design interventions that address provider bias, should it be ranked among the top influencers of facility choice. Understanding the interactions between the multiple factors that influence facility choice is important for determining how finite programmatic resources should be used. For example, it is necessary to determine the trade‐offs between focusing on process factors such as making more methods available or undertaking more community talks, compared to the alternative (or the addition) of training providers on age and parity biases that may affect women's access to and use of a full range of contraceptive methods.

## CONFLICTS OF INTEREST

The authors have no conflicts of interest to disclose.

## ETHICS APPROVAL STATEMENT

All study procedures, consent materials, and data collection tools for the household and facility‐based surveys were reviewed and approved by the Institutional Review Board at the University of North Carolina at Chapel Hill and the Comité National d'Ethique pour la Recherche en Santé in Senegal. All women provided written consent to participate in the survey.

## PATIENT CONSENT STATEMENT

All women provided written consent to participate in the survey.

## Data Availability

Data are available for download after an approval, which involves signing a data use agreement and providing brief information about intent of use. Information can be found at: https://doi.org/10.15139/S3/12KUC0.

## Footnotes

1
https://www.familyplanning2020.org/senegal#:~:text=2012‐,Senegal%20FP2020%20Commitment,of%20the%20family%20planning%20program.

2
https://www.shopsplusproject.org/sites/default/files/resources/Sources%20of%20Family%20Planning%20Senegal.pdf

3 This is the unweighted percentage and number of observations; see Table 1 for weighted percentage using a modern method.

## Appendix

**TABLE A1:** Multivariate coefficients (SE) for choice of a facility for family planning with consent bias, marital status bias, any age bias, and any type of bias

	Marriage bias		Consent bias		Any type of age bias		Any bias	
	Coef.	SE	Coef.	Coef.	Coef.	SE	Coef.	SE
Bias measure	−0.3606	0.1822^*^	0.4416	0.1777^*^	−0.4458	0.0897^***^	−0.2702	0.0817^***^
Average distance	−0.9980	0.0647^***^	−0.9979	0.0648^***^	−1.0006	0.0646^***^	−0.9986	0.0644^***^
Open seven days a week	−0.0271	0.0712	−0.0397	0.0710	−0.0257	0.0702	−0.0383	0.0704
Average number of midwives	0.0042	0.0107	−0.0010	0.0106	0.0064	0.0106	0.0066	0.0107
Average number of clients last 30 days	0.0018	0.0003^***^	0.0020	0.0003^***^	0.0018	0.0003^***^	0.0018	0.0003^***^
Facility age	0.0062	0.0016^***^	0.0068	0.0015^***^	0.0058	0.0015^***^	0.0061	0.0015^***^
Facility does community talks/outreach	0.1366	0.0685^*^	0.1212	0.0687^+^	0.1413	0.0678^*^	0.1476	0.0679^*^
Facility had a social worker	0.4468	0.1190^***^	0.4189	0.1216^***^	0.4268	0.1209^***^	0.4328	0.1207^***^
Average number of methods available	0.0723	0.0266^**^	0.0717	0.0265^**^	0.0801	0.0273^**^	0.0786	0.0270^**^
Stock out pill or injectable in the last year	−0.1467	0.1812	−0.0629	0.1781	−0.1053	0.1786	−0.1424	0.1815
Private facility	−1.6293	0.2069^***^	−1.6671	0.2114^***^	−1.5850	0.2096^***^	−1.5899	0.2087^***^
Number of observations in model	152,681		152,681		152,681		152,681	

NOTE: Sample size for analysis is the total number of facilities that the sample of women could potentially access in their city. The coefficients show whether or not the corresponding variable is given positive or negative weight when choosing a facility. The standard errors are cluster corrected for multiple observations on the same respondent since each respondent is linked to all facilities.

^+^ p ≤ 0.10, ^*^p ≤ 0.05, ^**^p ≤ 0.01, ^****^p ≤ 0.001.

**TABLE A2:** Summary comparison of results (willingness to travel) from models of consent bias, marriage bias, any age bias, and any overall bias presented in Table A1

	Willingness to travel in kilometers for avoiding bias (95% confidence interval in parentheses)			
Group	Marriage bias/distance	Consent bias/distance	Any type of age bias/distance	Any bias/distance
All ages	0.3613 (‐0.0010, 0.7235)	−0.4426 (−0.7903, −0.0949)	0.4455 (0.2699, 0.6211)	0.2705 (0.1106, 0.4305)
<26	0.5284 (NS) (−0.2234, 1.2803)	−0.5876 (NS) (−1.2995, 0.1242)	0.4009 (−0.0022, 0.8040)	0.3030 (−0.0607, 0.6667)
26+	0.3232 (NS) (−0.0825, 0.7289)	−0.4220 (−0.8134, −0.0306)	0.4449 (0.2514, 0.6384)	0.2544 (0.0775, 0.4312)

NOTE: Models control for: distance, whether open all days of the week, number of midwives, number of new or continuing family planning clients, facility age, whether the facility does community talks, whether there is a social worker at the facility, the number of methods available, if there has been a stock out of pills or injectables in the last year, if it is a private facility, and the bias measure. (NS) indicates that the result is not significant at p ≤ 0.10.
